# Proteomics-based identification of haptoglobin as a favourable serum biomarker for predicting long-term response to splenectomy in patients with primary immune thrombocytopenia

**DOI:** 10.1186/1479-5876-10-208

**Published:** 2012-10-07

**Authors:** Chao-Xu Zheng, Zhuang-Qi Ji, Long-Juan Zhang, Qiong Wen, Liu-Hua Chen, Jun-Feng Yu, Dong Zheng

**Affiliations:** 1 Department of Minimal Invasive Surgery, The First Affiliated Hospital, Sun Yat-sen University, Guangzhou, 510080, China; 2 Laboratory of Surgery, The First Affiliated Hospital, Sun Yat-sen University, Guangzhou, 510080, China; 3 Department of Medicine, The First Affiliated Hospital, Sun Yat-sen University, Guangzhou, 510080, China; 4 Department of Haematology, The First Affiliated Hospital, Sun Yat-sen University, Guangzhou, 510080, China

**Keywords:** Primary immune thrombocytopenia, Splenectomy, Proteomics, Biomarkers, Haptoglobin

## Abstract

**Background:**

Splenectomy is the most effective treatment for patients with primary immune thrombocytopenia (ITP) who fail to respond to steroid therapy. Thus far, there is no effective means to predict the long-term haematological response of the procedure. The purpose of this study was to identify serum biomarkers as predictors of long-term response based on a proteomics approach.

**Methods:**

The serum samples of ITP patients were collected before splenectomy and seven days after surgery. After depletion of the abundant serum proteins, pooled preoperative serum samples from four responders to splenectomy, four nonresponders and four healthy controls were subjected to two-dimensional gel electrophoresis (2-DE). Nine protein spots with at least a five-fold alteration in expression between responders and nonresponders were all identified as haptoglobin (Hp) by matrix-assisted laser desorption/ionisation time-of-flight (MALDI-TOF) mass spectrometer (MS) analysis. The validation of serum Hp expression was performed using enzyme-linked immunosorbent assays (ELISA) in thirty-seven responders, thirteen nonresponders and twenty-one healthy controls.

**Results:**

The preoperative serum levels of Hp in the nonresponders (925.9 ± 293.5 μg/ml) were significantly lower than those in the responders (1417.4 ± 315.0 μg/ml, *p* <0.001) and the healthy controls (1409.1 ± 354.2 μg/ml, *p* <0.001), while there was no significant difference between the latter two groups. The postoperative serum levels of Hp in responders and nonresponders were (1414.1 ± 225.0 μg/ml) and (952.9 ± 202.4 μg/ml), respectively. There were no significant differences between the serum Hp levels before and after surgery in both responders and nonresponders (*p*>0.05). The preoperative serum levels of Hp did not significantly correlate with preoperative platelet count of the same blood samples (r = 0.244, *p* = 0.087), while it positively correlated with postoperative peak platelet count (r = 0.622, *p* < 0.001). The optimal cutoff value of preoperative serum Hp levels (1173.80 μg/ml) derived from the receiver operating characteristic (ROC) curve led to 78.4% sensitivity and 84.6% specificity.

**Conclusions:**

These results suggest that serum Hp levels may serve as a favourable predictor for the long-term response to splenectomy in ITP and may help to understand the pathophysiological differences between responders and nonresponders.

## Background

Primary immune thrombocytopenia (ITP), also known as primary immune thrombocytopenic purpura or idiopathic thrombocytopenic purpura, is an immune-mediated acquired disease characterised by low platelet counts and bleeding complications in children and adults [1]. Patients with platelet counts persistently lower than 30 × 10^9^/L are at risk for life-threatening bleeding. The main mechanisms of ITP are anti-platelet autoantibody-mediated platelet destruction and inhibition of thrombopoiesis [2]. Other mechanisms such as abnormal T-helper cells type 1 (Th1) and type 2 (Th2) responses, complement activation, and direct T-cell cytotoxicity were also found in ITP patients [2].

The spleen has been considered the main site for the autoimmune processes in ITP pathophysiology [3]. Splenectomy has proven to be a preferred treatment for ITP patients who do not respond to glucocorticoid treatment or continue to need high-dose glucocorticoids to maintain a safe platelet count. The results of numerous studies indicate that approximately two-thirds of adult patients and 70 to 80 percent of children achieve a durable response to splenectomy [1]. However, splenectomy for ITP is often associated with the risk of major morbidity and mortality, and the long-term haematological outcomes of the procedure cannot be predicted through routinely available measures [4]. Some studies reported that several variables may predict a stable response to splenectomy including younger age [5-7], previous response to steroids [6,7] and postoperative peak platelet count [8,9]. Other studies showed opposite results [10-12]. Najean et al. found the site of autologous ^111^In-labeled platelet sequestration to be a good prognostic parameter [13]. However, the techniques of isotope assessments are often qualitative, rather than quantitative, and many patients with nonsplenic sequestration also respond well to splenectomy [4]. Hence, the assessments of platelet sequestration are not widely accepted. The fear of failure to respond to the splenectomy renders many patients reluctant to undergo surgery and haematologists hesitant to recommend the surgery. Therefore, it is imperative to identify a preoperative serum biomarker that can serve as a predictor of good response to splenectomy and help patients and physicians with the decision of using surgery to treat ITP.

In this study, we used two-dimensional gel electrophoresis (2-DE) technology coupled with matrix-assisted laser desorption/ionisation time-of-flight (MALDI-TOF) mass spectrometer (MS) analysis to identify differentially expressed proteins in ITP patients with different responses to splenectomy. The identified proteins were further confirmed by enzyme-linked immunosorbent assays (ELISA) to evaluate their values in predicting the long-term response to splenectomy.

## Methods

### Patients and healthy controls

This study included fifty-eight patients (twenty males and thirty-eight females; mean age 38.1 ± 15.6 years; age range 16–70 years) undergoing laparoscopic splenectomy for chronic ITP at the Department of Minimal Invasive Surgery, the First Affiliated Hospital of Sun Yat-sen University, between May 2007 and April 2011. All patients recovered well without any major postoperative morbidity. The diagnosis of ITP was made according to the guidelines published by the British Committee for Standards in Haematology General Haematology Task Force [14]. Other possible causes of thrombocytopenia were ruled out. Routine blood tests (including haemoglobins, reticulocyte counts, bilirubin levels, and lactate dehydrogenase) were performed for all patients enrolled in this study. Direct Coombs testings had been done in the patients presenting with anaemia. No evidence of haemolysis was found in these patients and Evans syndrome was excluded from this corhort. The indications for splenectomy in these patients included those did not respond to steroids at all and/or the requirement of prolonged use of high dose steroids to maintain a platelet count over 30×10^9^/L. All patients received corticosteroids or immunosuppressive agent treatment until they underwent laparoscopic splenectomy. The dosages of steroids were then reduced gradually after surgery. The blood samples of ITP patients were collected before splenectomy and seven days after surgery. None of the patients enrolled in this study received any platelet transfusions, intravenous immunoglobulin or anti-RhD immunoglobulin (anti-D) treatments for at least two weeks before the collection of the preoperative blood samples. Twenty-five age- and sex-matched healthy subjects served as the controls. The study was approved by the Ethical Committee of the First Affiliated Hospital of Sun Yat-sen University. Informed consent was obtained from each patient and healthy subject.

### Definition of long-term response to splenectomy

All of the patients were followed up after splenectomy throughout the study. In this study, we adopted the definition of haematological outcome suggested recently by the international working group [1]. Complete response (CR) was defined as a platelet count ≥ 100×10^9^/L and the absence of bleeding. Response (R) is defined as any platelet count between 30 and 100×10^9^/L and at least a two-fold increase in the baseline count and the absence of bleeding. No response (NR) is defined as any platelet count lower than 30×10^9^/L or less than a two-fold increase in the baseline platelet count or the presence of bleeding. In this study, the long-term response to splenectomy was defined as a complete response or response from twelve months to the last follow-up without any medications. For the statistical analysis, the patients were subdivided into the responder (CR+PR) group (n=41) and the nonresponder (NR) group (n=17) according to the long-term follow-up results.

### Serum sample preparation

Fresh whole blood samples were collected during a fasting state in the early morning. All samples were transported on ice and centrifuged immediately at 3000 g for 15 min at 4°C. The supernatants were divided into 500 μl aliquots and stored at −80°C until further analysis. Blood samples from four patients in the responder group, four in the nonresponder group, and four healthy controls were used for the screening study. The serum samples from the other thirty-seven responders, thirteen nonresponders, and twenty-one healthy controls were used for the validation study.

Highly abundant proteins in serum were depleted using a Proteoprep Blue Albumin and IgG Depletion Kit (Sigma-Aldrich, Missouri, USA) according to the manufacturer’s instructions. The final protein concentration was detected using the Bio-Rad Protein Assay technique.

### Two-dimensional electrophoresis and image analyses

Isoelectric focusing (IEF) was performed on an Ettan IPGphor III IEF System (Amersham Bioscience, Uppsala, Sweden) at 20°C. Immobiline DryStrips (18 cm long, pH 4–7) (Amersham Bioscience, Uppsala, Sweden) were rehydrated actively at 30 V for 12 h with 750 μg of depleted serum protein that was premixed with rehydration buffer containing 7 M urea, 2 M thiourea, 2% 3-[(3-cholamidopropyl) dimethylammonio]-1-propanesulfonate (CHAPS), 0.5% (v/v) ampholytes (pH 4–7), 20 mM dithiothreitol (DTT) and a trace of bromophenol blue (making the final volume of 350μl). IEF was conducted with gradient voltages of 200 V for 1 h, 1000 V for 1 h, a 3 h linear ramp up to 10,000 V, and final focusing at 10,000 V for a total of 110,000 Vh. The immobilized pH gradient (IPG) strips were equilibrated in buffer 1 (75 mM Tris–HCl, pH 8.8, 6 M urea, 30% (v/v) glycerol, 2% (w/v) sodium dodecyl sulfate (SDS), 1% (w/v) DTT and a trace of bromophenol blue) for 15 min and then subsequently alkylated in buffer 2 (75 mM Tris–HCl pH 8.8, 6 M urea, 30% (v/v) glycerol, 2% (w/v) SDS, 2.5% (w/v) iodoacetamide and a trace of bromophenol blue) for 15 min. The IPG strip was then transferred to a 12.5% SDS polyacrylamide gel, and the second dimensional separation was performed in an Ettan DALT six Electrophoresis Unit (Amersham Bioscience, Uppsala, Sweden) with the power set at 1 w/gel for 1 h followed by 15 w/gel until the tracking dye migrated to within 1 cm of the bottom of the gel at 15°C. The gels were stained with colloidal coomassie as described in the manual.

The 2-DE gel images were analysed using Image Master 2D Platinum 6.0 software (Amersham Bioscience, Uppsala, Sweden). The relative volumes of spots were used for comparison between two patient groups. Our study mainly focused on the highly differentially expressed proteins whose expressions were strongly associated with response to splenectomy in ITP patients. As described by Zhang et al. [15], the criterion for highly differentially expressed protein was also set as at least a five-fold change in spot volume between the two matched sets in our study. Only those highly differentially expressed protein spots (at least five-fold alterations in expression in responders versus nonresponders) were chosen for further analysis by MALDI-TOF/TOF MS.

### In-gel digestion and MALDI-TOF/TOF MS analyses

Protein spots of interest were excised manually from 2-DE gels, destained with 15 mM potassium ferricyanide and 50 mM sodium thiosulfate, and subsequently dehydrated in 100% acetonitrile. Each sample was then treated with 2 μl (25 ng/μl) of modified porcine trypsin in 25 mM ammonium bicarbonate (pH 8) overnight at 37°C. The trypsin solutions were collected for mass spectrometry analysis.

Protein identification was performed with an Ultraflex III mass spectrometer (Bruker Daltonics, Bremen, Germany) and operated in the positive ion reflectron mode. A saturated solution of α-cyano-4-hydroxycinnamic acid in 50% acetonitrile and 0.1% trifluoroacetic acid was used as the matrix. A standard peptide mixture with a mass range 800–4000 Da (Bruker Daltonics, Germany) was used for external calibration. The subsequent MS/MS analysis was performed in a data-dependent manner, and the five most abundant ions fulfilling certain pre-set criteria were subjected to LIFT for a post-source decay analysis.

Peptide mass fingerprints (PMFs) and MS/MS analyses were searched by the BioTools software (version 3.0, Bruker Daltonics, Germany) against the SwissProt protein database. The search parameters were as follows: trypsin digestion with maximum one missed cleavage; carbamidomethylation of cysteine as fixed modification and oxidation of methionine as variable modification; peptide mass tolerance, ± 100 ppm; fragment mass tolerance, ± 0.5 Da; peptide charge, +1; and monoisotopic mass. Protein identifications were accepted when the peptide score was higher than the threshold value (*p*<0.05), and manual interpretation was used to confirm agreement between the spectra and peptide sequence.

### Validation of identified proteins by ELISA

To validate the haptoglobin (Hp) expression in serum, serum samples from ITP patients and healthy controls were analysed by ELISA. The Hp levels of serum samples collected seven days after surgery were also evaluated at the same time. The concentrations of Hp in serum were determined using an AssayMax human Hp ELISA kit according to the manufacturer's protocol (Assaypro, Winfield, MO, USA).

### Statistical analysis

Statistical analysis was carried out using SPSS 16.0 for Windows software (SPSS Inc., Chicago, IL, USA). Comparisons of gender and previous response to steroids between two patient groups were carried out using the chi-square test. Continuous variables were expressed as mean ± standard deviation (SD). Comparisons were performed using the Mann–Whitney test between two independent groups, or one-way ANOVA followed by the least-significant difference test for three groups. The changes of the serum Hp levels before and after surgery in responders and nonresponders were analysed with Wilcoxon test. Correlation between parameters was assessed by calculating the Pearson’s correlation coefficient. Receiver operating characteristic (ROC) curve was used to evaluate the predictive value of preoperative serum levels of Hp for long-term haematological outcomes of surgery. The area under the curve (AUC) with a 95% confidence interval (CI) was computed. Sensitivity and specificity were calculated according to different cutoff values. A *P* value less than 0.05 was considered statistically significant.

## Results

### Study population

Table 1 summarises the baseline clinical characteristics of fifty-eight patients. There were no significant differences in the age, gender, previous response to steroids, disease duration before the surgery, preoperative platelet count or the duration of follow-up between the responder and nonresponder groups. However, a significantly higher postoperative peak platelet count was observed in the responders compared to the nonresponders (*P*<0.001).

**Table 1:** Baseline Characteristics of Patients

**Variables**	**Responder group (n=41)**	**Nonresponder group (n=17)**	***P *value**
Age, year	36.4 ± 14.9	42.3 ± 16.9	0.203
Gender (male/female)	13/28	7/10	0.490
Previous response to steroids (response/resistance)	21/20	5/12	0.128
Disease duration, months	35.9 ± 34.1	30.9 ± 31.4	0.851
Preoperative platelet count (×10^9^/L)	29.9 ± 28.4	18.0 ± 10.4	0.090
Postoperative peak platelet count (×10^9^/L)	439.9 ± 171.0	201.1 ± 132.6	<0.001
Follow-up, months	34.4 ± 13.4	35.3 ± 13.9	0.824

### Identification of Hp by 2-DE separation and MALDI-TOF/TOF-MS analyses

**Figure 1:**
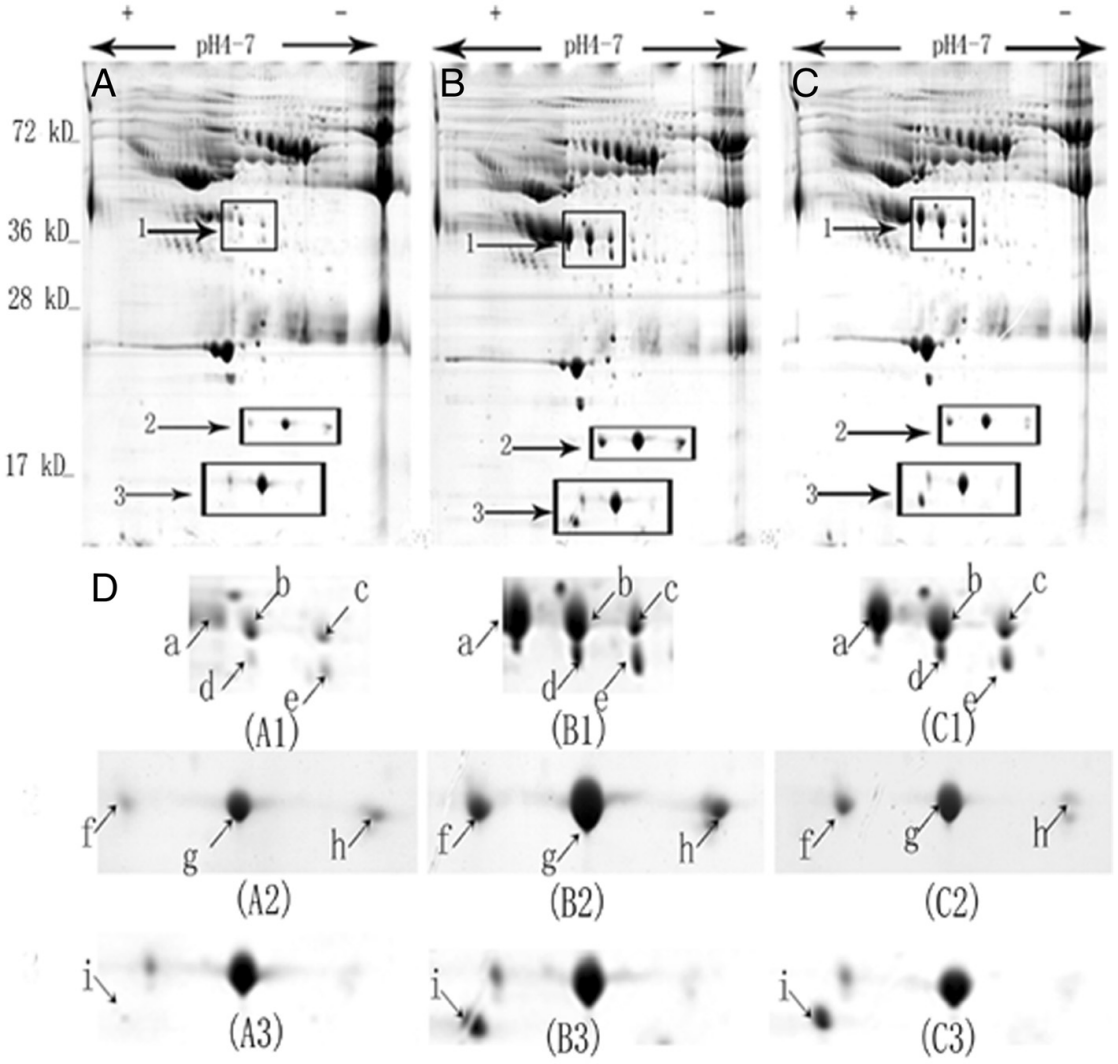
**Differentially expressed protein spots identified by 2-DE image analysis.** The original 2-DE images representing all protein spots present in serum samples from the nonresponder group (**A**), the responder group (**B**), and the healthy control group (**C**). The enlarged images (**D**) showing the magnified regions 1–3 of the corresponding 2-DE gels. Nine identified protein spots (a-i, arrows showing the corresponding locations) with significantly differential expressions among the three groups were visualised. Protein spots a to i showed obviously decreased expressions in the nonresponders compared with the responders and healthy controls.

**Figure 2:**
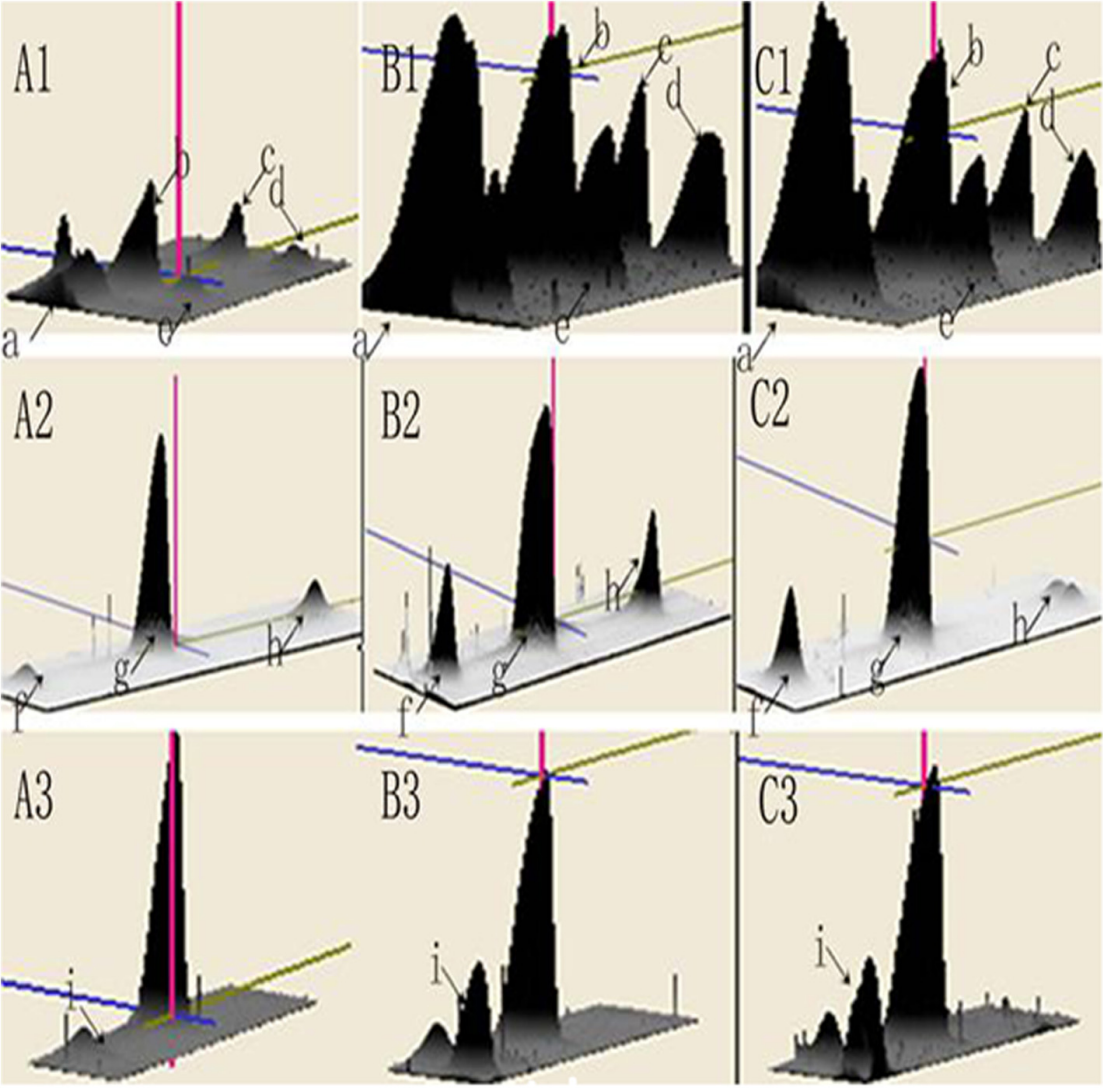
**3-D view of highly differentially expressed proteins.** The computational analysis of the images with Image Master 2D Platinum 6.0 software allowed for the detection of protein volumes. Significant changes of more than 5-fold were found in the protein spots (spots a to i) of the magnified regions 1–3 of the corresponding 2-DE gels among the nonresponder group (**A**), the responder group (**B**), and the healthy control group (**C**). The differentially expressed proteins were down-regulated in the nonresponders compared with the responders and healthy controls.

Pooled preoperative serum proteins from four patients in the responder group, four in the nonresponder group, and four healthy controls were subjected to 2-DE separation. Two-dimensional electrophoresis was performed four times per sample to minimise gel-to-gel variations. Figure 1A, 1B, and 1C show original 2D gel images for all samples of the three groups and identify a number of distinct protein spots. The enlarged images of regions 1–3 in the corresponding 2-DE gels reveal significant differential changes for some of the potential novel biomarkers (spots a-i, Figure 1D). By quantitative comparison of spot volumes, nine protein spots (spots a-i, Figure 2) were found to be down-regulated more than five-fold in the nonresponders when compared with the responders.

**Figure 3:**
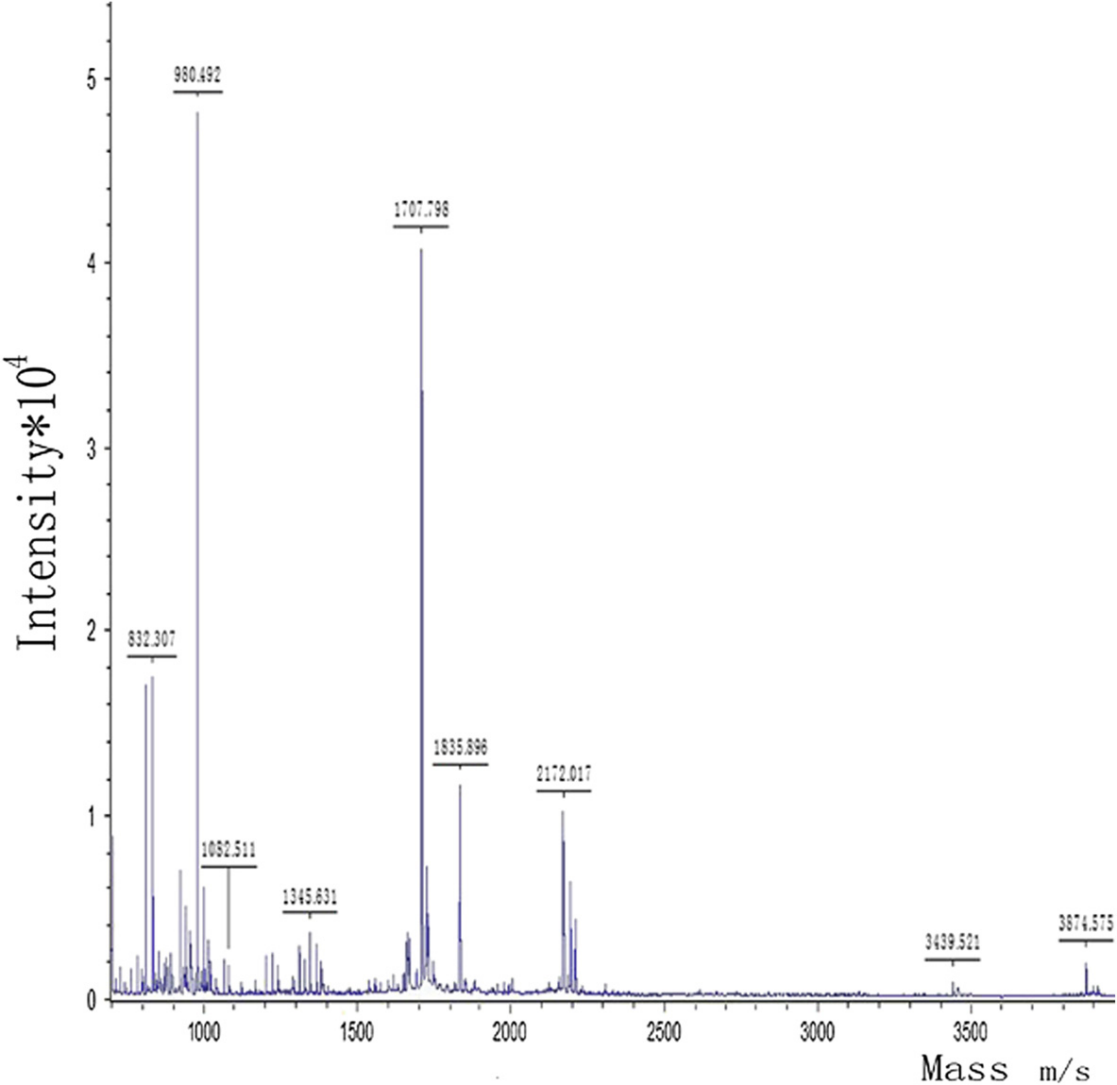
**Representative MALDI-TOF peptide mass fingerprint spectrum of spot b.** After trypsin digestion, the resulting peptides were analysed by MALDI-MS and MS/MS analyses. Peptide mass fingerprints (PMFs) data were searched against the database of SwissProt by BioTools 3.0 software. The protein with molecular mass of approximately 42 kDa was identified as haptoglobin (Hp) by the sequence analysis.

These nine protein spots were then excised from the gels and analysed by MALDI-TOF/TOF-MS. The results revealed that all of these protein spots were identified as Hp with different isoelectric points (p*I*) and molecular weights (MW) (Table 2). Figure 3 is a representative MALDI-TOF peptide mass fingerprint spectrum of trypsin-digested spot b, which was remarkably down-regulated in the nonresponders and was later identified as Hp.

**Table 2:** Identification of differentially expressed proteins by MALDI-TOF/TOF

**Spot number**	**Protein description**	**Accession number**	**MS method**	**MASCOT score**	**Matched peptides**	**Sequence coverage, %**	**MW (kD)/p*I***	**Fold change (responder/ nonresponder, n=4)**
a	Haptoglobin	P00738	MS,MS/MS	84,92	12,2	34%,5%	42.1/5.08	8.11
b	Haptoglobin	P00738	MS,MS/MS	67,116	12,3	33%,8%	42.2/5.21	5.94
c	Haptoglobin	P00738	MS,MS/MS	103,90	10,2	32%,6%	42.0/5.37	5.50
d	Haptoglobin	P00738	MS,MS/MS	92,100	12,2	32%,6%	39.1/5.21	5.60
e	Haptoglobin	P00738	MS,MS/MS	109,66	10,2	32%,6%	38.7/5.38	7.30
f	Haptoglobin	P00738	MS/MS	46	2	6%	16.9/5.40	9.57
g	Haptoglobin	P00738	MS/MS	129	1	6%	17.1/5.68	5.21
h	Haptoglobin	P00738	MS/MS	94	2	7%	16.9/6.07	5.35
i	Haptoglobin	P00738	MS/MS	54	1	3%	11.9/5.13	16.50

### Decreased expression of Hp in the serum of nonresponders

**Figure 4:**
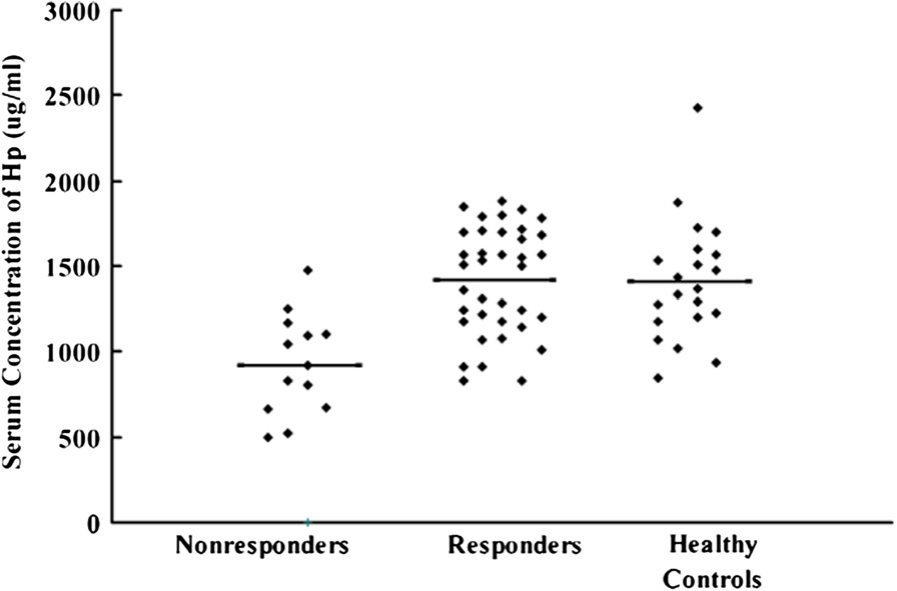
**The preoperative serum level of Hp was validated by ELISA.** One-way ANOVA analysis showed significant difference of preoperative serum Hp expressions among the three groups. The preoperative serum levels of Hp in thirteen nonresponders was significantly lower than that of thirty-seven responders (*p* <0.001) and twenty-one healthy controls (*p* <0.001). No significant difference was seen between the responders and healthy controls (*p* > 0.05).

ELISA was further performed to validate the results of the 2-DE. As shown in Figure 4, the preoperative serum levels of Hp in thirty-seven responders, thirteen nonresponders, and twenty-one healthy controls were (1417.4 ± 315.0 μg/ml, 95% CI: 1312.3-1522.4 μg/ml), (925.9 ± 293.5 μg/ml, 95% CI:748.5-1103.2 μg/ml) , and (1409.1 ± 354.2 μg/ml, 95% CI: 1247.9-1570.3 μg/ml), respectively. One-way ANOVA analysis showed that the preoperative serum levels of Hp in the nonresponders were significantly lower than those in the responders (*p* < 0.001) and healthy controls (*p* < 0.001). There was no significant difference in the preoperative serum levels of Hp between the responders and healthy controls (*p* > 0.05). The postoperative serum levels of Hp in the responders and the nonresponders were (1414.1 ± 225.0 μg/ml, 95% CI: 1339.1-1489.1 μg/ml) and (952.9 ± 202.4 μg/ml, 95% CI: 830.6-1075.2 μg/ml), respectively. There were no significant differences between the serum Hp levels before and after surgery in both responders and nonresponders (*p*>0.05). Pearson’s correlation analysis showed that the preoperative serum levels of Hp did not significantly correlate with preoperative platelet count of the same blood samples collected for this study in ITP patients (r = 0.244, *p* = 0.087), while a positive correlation was found between the preoperative serum Hp levels and postoperative peak platelet count (r = 0.622, *p* < 0.001).

### Predictive value of preoperative serum levels of Hp

**Figure 5:**
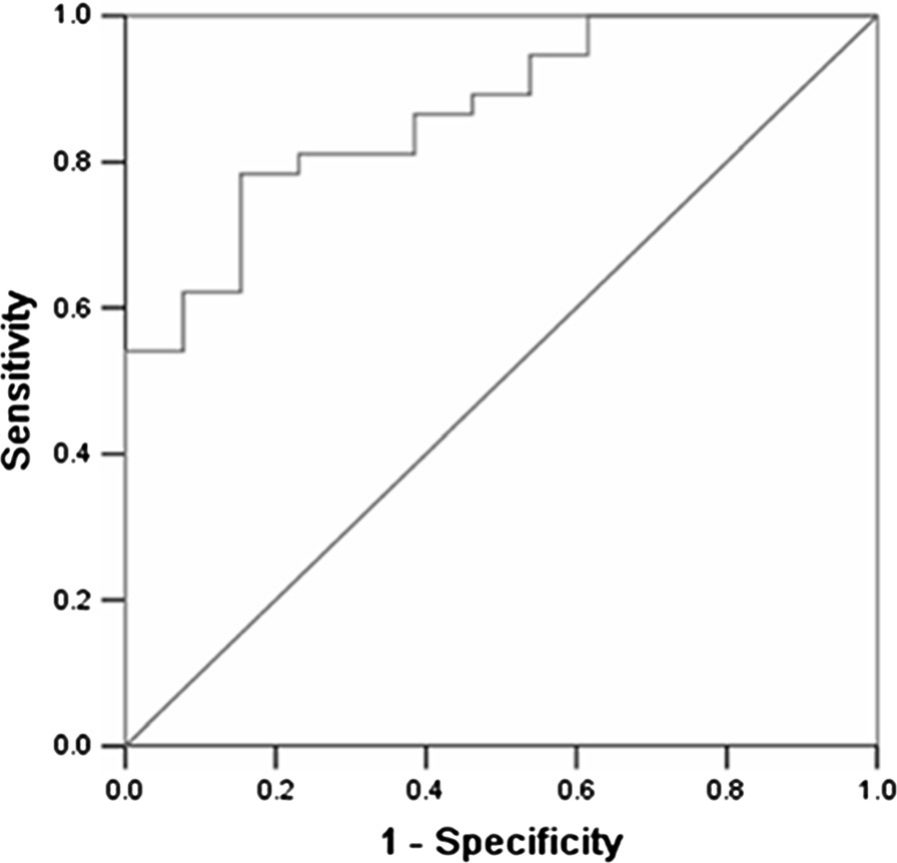
**Receiver operating characteristic (ROC) curve for the preoperative serum level of Hp in responders versus nonresponders.** The area under curve (AUC) value of the comparison between groups was 0.867 (95% CI: 0.764-0.970). The optimal cutoff value of preoperative serum Hp levels (1173.80 μg/ml) derived from the ROC curve led to 78.4% sensitivity and 84.6% specificity.

Using the ROC curve method, we evaluated the predictive value of the preoperative Serum Hp levels for long-term response to splenectomy. As showed in Figure 5, AUC was 0.867 (95% CI: 0.764-0.970), indicating that the preoperative Serum levels of Hp was a good predictor to differentiate responders from nonresponders. Table 3 showed the sensitivity and specificity of different cutoff values. Based on the maximum Youden index (sensitivity+specificity-1), the optimal cutoff value from the ROC curve, Hp ≥ 1173.80 μg/ml, was chosen. When this value was set, the sensitivity was 78.4% and the specificity was 84.6% for discriminating responders from nonresponders.

**Table 3:** Sensitivity and specificity for the preoperative serum Hp levels at different cutoff values

**Cutoff (μg/ml)**	**Sensitivity (%)**	**Specificity (%)**	**Youden index**
816.42	100	38.5	0.385
911.41	91.9	46.1	0.380
1026.61	86.5	53.8	0.403
1073.74	83.8	61.5	0.453
1119.61	81.1	76.9	0.580
1173.80	78.4	84.6	0.630
1265.94	62.2	92.3	0.545
1485.29	54.1	100	0.541

## Discussion

Comprehensive analysis of the changes in serum proteomes are an important part of broad evaluations of the diagnosis, prognosis and therapeutic response of progressing diseases, such as autoimmune disorders, cardiovascular diseases, and cancers. With commonly used proteomic approaches, it is feasible to discover differentially expressed proteins in samples from patients that are likely to be involved in the autoimmune process or serve as biomarkers that correlate with disease or therapeutic outcomes [16,17]. To date, only one proteomic-based study for biomarkers has been reported in ITP patients. In the study, one-dimensional gel electrophoresis was used to separate serum proteins in ITP patients, non-ITP patients with thrombocytopenia and healthy controls [18]. Quantification of the serum proteins identified by liquid chromatography-tandem mass spectrometric (LC-MS/MS) analysis showed that the serum ceruloplasmin levels in ITP patients were statistically significantly higher than in non-ITP patients and controls. For the purpose of identifying the potential biomarkers predicting long-term response to splenectomy in ITP patients, the present study carried out a comparative proteomics analysis with 2-DE and MS on the sera from the responders, nonresponders and healthy controls. Nine protein spots that were differentially expressed by at least five-fold between the responder and nonresponder samples were further analysed with MS/MS. All of the spots were identified as Hp by the peptide sequence analysis. Although the measurement of serum ceruloplasmin levels was found to be useful for the diagnosis of ITP in the report mentioned above, we did not identify ceruloplasmin as a highly differentiated expressed protein between responders and nonresponders in this study.

Hp is an acute phase protein that is synthesised predominately in the liver by hepatocytes. The major inducer of the expression of Hp is IL-6, which is produced through the activities of the primary cytokines TNF-α and IL-1 [19]. The serum levels of Hp remains fairly constant in any given individual; therefore, the observation of a marked change in serum Hp expression has clinical significance [20]. Hp consists of two different polypeptide chains, α chain and β chain. There is only one type of β chain with a MW of approximately 40 kDa. However, the α chain is represented by two isoforms, α-1 and α-2, with the α-1 isoform being approximately 9 kDa and the α-2 being 16 kDa in MW [21]. After chemical reduction of Hp, these polypeptide chains can be clearly revealed in the images of gel electrophoresis [21]. In this study, five protein spots with MW of 38.7 - 42.2 kDa, shown in region 1 of the 2-DE images, were identified as the β chain of Hp. While three protein spots in region 2 (MW 16.9 - 17.1 kDa) and one protein spot in region 3 (MW 11.9 kDa) represented the α-2 and α-1 isoforms of Hp.

Hp has been proven to modulate both innate and adaptive immune responses in several aspects. Th1 and Th2 play a crucial role in the pathogenesis of autoimmune diseases [22]. Studies performed both *in vitro* and *in vivo* indicate that Hp exhibits a significant modulating impact on the Th1/Th2 balance via an inhibitory effect on Th2 cytokine release and therefore promotes a dominant Th1 cellular response [23,24]. Hp supports proliferation and functional differentiation of B and T cells as part of homeostasis and in response to antigen stimulation. Analysis of blood from Hp-deficient mice showed a significant reduction in B and T lymphocytes, and the relative number of splenic monocytes and dendritic cells appeared elevated with respect to wild-type mice [25]. A more recent study showed that Hp-deficient mice facilitate the development of autoimmune disease of central nervous system, which is associated with the enhanced production of several inflammatory cytokines in the spinal cord [26]. Hp is characterised by molecular heterogeneity, with three major phenotypes: Hp 1–1, Hp 2–2, and the heterozygous Hp 2–1. The Hp 2–2 genotype was associated with decreased serum concentrations of Hp. A significant over-representation of the Hp 2–2 genotype has been shown in human immune disorders, such as rheumatoid arthritis, diabetes mellitus type 2, and inflammatory bowel diseases [27]. Taken together, these studies suggest that Hp may play an important role in the pathogenesis and clinical course of autoimmune diseases through various immunomodulatory effects. To date, very few studies in the current literature focused on the issue of the serum Hp expression in ITP patients. A recent study found that abnormally low levels of free serum Hp were present in eleven out of thirty paediatric patients with ITP [28]. The authors considered Hp as a sensitive indicator of haemolysis and presumed that patients with low levels of serum Hp may develop autoimmune haemolytic anaemia in long-term follow-up studies. Until now, the pathophysiological roles of Hp in ITP were poorly understood.

Recent proteomic studies showed that Hp served as an important biomarker in some autoimmune diseases. Increased protein levels of Hp were found in the cerebrospinal fluid from patients with Guillain-Barré syndrome, which is an acute inflammatory autoimmune disorder in the peripheral nervous system [29,30]. Other studies reported that elevated serum levels of Hp correlated with higher disease activity in systemic lupus erythematosus and active Behcet’s disease [31,32]. In the present study, we studied the differentially expressed proteins in the serum of ITP patients with different responses to splenectomy using a comparative proteomics approach. Hp was identified as a highly differentially expressed protein between nonresponders and responders. Further validation by ELISA showed that the preoperative serum levels of Hp in the nonresponders were significantly lower than those in the responders and healthy controls. No statistical differences of preoperative serum Hp expression were observed between the responders and healthy controls. The results indicate that the preoperative serum levels of Hp may serve as an important predictor of long-term haematological response to splenectomy in ITP patients. Patients with lower preoperative serum levels of Hp tend to have a worse response to surgery when compared with those with higher preoperative serum levels. The ROC curve is a useful method for evaluating clinical usefulness of a biomarker. The ROC curve analysis showed that AUC of the preoperative serum Hp levels reached 0.867, which means that the preoperative serum Hp levels can be used as a potential predictive biomarker. The Youden index has been demonstrated to be a preferred means of identifying the optimal cutoff value from the ROC curve[33]. Therefore the optimal cutoff value of the preoperative serum Hp levels (1173.80 μg/ml) was identified based on the the maximum Youden index in this study. At this cutoff value, the sensitivity was 78.4% and the specificity was 84.6%. The present study also found that the serum Hp levels did not change seven days after surgery in both responders and nonresponders. To the best of our knowledge, this is the first paper reporting a potential serum biomarker for predicting the long-term response to splenectomy in ITP patients. However, potential limitations of this study must be considered. While the removal of abundant serum proteins would facilitate the discovery and detection of less abundant proteins, including disease associated markers. It should be noted that this technique remains controversial in biomarker discovery, as it may also remove other possibly more interesting proteins bound to the abundant proteins[34]. Since relapses of ITP may occur even two years after splenectomy, further studies with larger sample size and long-term follow-up are needed to confirm our findings. Evans syndrome which links to low serum level of Hp and poor response to splenectomy should be strictly excluded from the study.

## Conclusions

Our previous study found that the postoperative peak platelet count was closely correlated with the long-term response of splenectomy in ITP patients [35]. Similar results were also shown in this study. However, this variable can only be obtained after surgery and is of no use for making the decision of therapeutics. The present study demonstrates that by using a proteomic approach, we are able to identify Hp as a potential serum biomarker, which may serve as a major predictor of long-term response to splenectomy in ITP patients. The preoperative serum levels of Hp were also shown to be positively correlated with postoperative peak platelet count in ITP patients. These findings may be critical for providing new insights into the pathogenic mechanisms of ITP and optimising the strategic choice of surgical intervention. Further research investigating the biology of Hp would help to understand the pathophysiological differences between responders and nonresponders.

## Abbreviations

ITP: Primary immune thrombocytopenia; 2-DE: Two-dimensional gel electrophoresis; MALDI-TOF: Matrix-assisted laser desorption/ionisation time-of-flight; MS: Mass spectrometer; Hp: Haptoglobin; ELISA: Enzyme-linked immunosorbent assays; IEF: Isoelectric focusing; DTT: Dithiothreitol; IPG: Immobilized pH gradient; SDS: Sodium dodecyl sulphate; PMFs: Peptide mass fingerprints; p*I*: Isoelectric points; MW: Molecular weights.

## Competing interests

The authors declare that they have no competing interests.

## Authors’ contributions

ZCX and JZQ performed the most of the experiments. ZLJ assisted in experimental design and helped to interpret data.WQ participated in acquiring laboratory data analysis. CLH and YJF collected samples and helped to interpret data. ZCX and ZD designed research and wrote the manuscript. All authors read and approved the final manuscript.
